# Nutritional risk assessment in critically ill cancer patients: systematic review

**DOI:** 10.5935/0103-507X.20150032

**Published:** 2015

**Authors:** Ana Valéria Gonçalves Fruchtenicht, Aline Kirjner Poziomyck, Geórgia Brum Kabke, Sérgio Henrique Loss, Jorge Luiz Antoniazzi, Thais Steemburgo, Luis Fernando Moreira

**Affiliations:** 1 Postgraduate Program in Medicine: Surgical Sciences, Faculdade de Medicina, Universidade Federal do Rio Grande do Sul - Porto Alegre (RS), Brazil.; 2 Intensive Care Unit, Hospital de Clínicas de Porto Alegre - Porto Alegre (RS), Brazil.; 3 Department of Nutrition, Universidade Federal do Rio Grande do Sul - Porto Alegre (RS), Brazil.

**Keywords:** Neoplasms/complications, Critically illness, Nutritional assessment, Nutritional status, Patient care, Intensive care

## Abstract

**Objective:**

To systematically review the main methods for nutritional risk assessment used in critically ill cancer patients and present the methods that better assess risks and predict relevant clinical outcomes in this group of patients, as well as to discuss the pros and cons of these methods according to the current literature.

**Methods:**

The study consisted of a systematic review based on analysis of manuscripts retrieved from the PubMed, LILACS and SciELO databases by searching for the key words “nutritional risk assessment”, “critically ill” and “cancer”.

**Results:**

Only 6 (17.7%) of 34 initially retrieved papers met the inclusion criteria and were selected for the review. The main outcomes of these studies were that resting energy expenditure was associated with undernourishment and overfeeding. The high Patient-Generated Subjective Global Assessment score was significantly associated with low food intake, weight loss and malnutrition. In terms of biochemical markers, higher levels of creatinine, albumin and urea were significantly associated with lower mortality. The worst survival was found for patients with worse Eastern Cooperative Oncologic Group - performance status, high Glasgow Prognostic Score, low albumin, high Patient-Generated Subjective Global Assessment score and high alkaline phosphatase levels. Geriatric Nutritional Risk Index values < 87 were significantly associated with mortality. A high Prognostic Inflammatory and Nutritional Index score was associated with abnormal nutritional status in critically ill cancer patients. Among the reviewed studies that examined weight and body mass index alone, no significant clinical outcome was found.

**Conclusion:**

None of the methods reviewed helped to define risk among these patients. Therefore, assessment by a combination of weight loss and serum measurements, preferably in combination with other methods using scores such as Eastern Cooperative Oncologic Group - performance status, Glasgow Prognostic Score and Patient-Generated Subjective Global Assessment, is suggested given that their use is simple, feasible and useful in such cases.

## Abstract (pt)

**Objetivo:**

Revisar sistematicamente os principais métodos para avaliação do risco nutricional utilizados em pacientes oncológicos graves e apresentar aqueles que melhor avaliam os riscos e preveem desfechos clínicos relevantes neste grupo de pacientes, além de discutir as vantagens e as desvantagens destes métodos, segundo a literatura atual.

**Métodos:**

O estudo consistiu de uma revisão sistemática com base na análise de artigos obtidos nas bases de dados PubMed, LILACS e SciELO, realizando as buscas com os termos em inglês: *“nutritional risk assessment”, “critically ill”* e *“cancer”*.

**Resultados:**

Apenas 6 (17,7%) dos 34 artigos inicialmente obtidos cumpriam os critérios para inclusão e foram selecionados para revisão. Os principais desfechos destes estudos foram que o gasto de energia em repouso se associou com subnutrição e superalimentação. O escore elevado da Avaliação Subjetiva Global - Produzida pelo Paciente associou-se de forma significante com baixa ingestão de alimentos, perda de peso e desnutrição. Em termos de marcadores bioquímicos, níveis mais elevados de creatinina, albumina e ureia se associaram de forma significante com mortalidade mais baixa. Os piores índices de sobrevivência foram encontrados para pacientes com condições de desempenho piores, conforme avaliação usando o *Eastern Cooperative Oncologic Group performance status*, escore prognóstico de Glasgow elevado, baixa albumina/ hipoalbuminemia, elevado escore da Avaliação Subjetiva Global - Produzida Pelo Paciente e para níveis elevados de fosfatase alcalina. Valores de avaliação do Índice de Risco Nutricional Geriátrico inferiores a 87 se associaram de forma significante com mortalidade. O escore pelo índice prognóstico inflamatório nutricional se associou com condição nutricional anormal em pacientes oncológicos graves. Dentre os estudos revisados que avaliaram apenas peso e índice de massa corporal, não se encontrou qualquer desfecho clínico significante.

**Conclusão:**

Nenhum dos métodos revisados ajudou a definir o risco entre esses pacientes. Portanto, sugere-se a avaliação por meio da quantificação da perda de peso e dos níveis séricos, preferivelmente em combinação com outros métodos utilizando escores como o Eastern Cooperative Oncologic Group performance status, o escore prognóstico de Glasgow e a Avaliação Subjetiva Global - Produzida Pelo Paciente, já que seu uso é simples, factível e útil em tais casos.

## INTRODUCTION

Cancer has been established as a public health problem worldwide and is currently the second leading cause of death due to disease in developed countries.^(1)^ Global rates are estimated to increase by 50% between 2000 and 2020, resulting in an incidence of 10 to 15 million cancer patients.^(2)^

It has been observed that patients with malignant diseases have been increasingly admitted to intensive care unit (ICU) due to complications from cancer itself or from side effects of therapy.^(3)^ Thus, a significant proportion of these cancer patients are indeed critically ill, leading to a significant increase in complications and death following treatment.^(4)^

Severe metabolic responses, mainly characterized by hypermetabolism and protein hypercatabolism, are present in critically ill patients, making them more susceptible to malnourishment.^(4)^ Malnourishment is linked to poor prognosis and should be detected and prevented as early as possible to treat and prevent clinical damage through appropriate and intensive nutritional intervention,^(5,6)^ which can reduce or even virtually abolish the risk of morbidity and mortality.^(7)^

One of the greatest problems with the currently available methods to assess nutritional status is the nearly absolute inadequacy of any method or tool used alone, clearly demonstrating the absence of a gold standard. Thus, different methods have been combined in an attempt to increase the specificity and sensitivity of nutritional risk assessment.^(8,9)^ Whether routine nutritional assessments in general or in cancer patients specifically already have many difficulties and do not allow establish a gold standard, the nutritional risk assessment of cancer patients which seriously complicate, becoming critical - as a direct result of cancer *per se* or as a result of the side effects of neo adjuvant or adjuvant therapy - should be estimated. The results will allow greater prevention of malnutrition, controlling risks and lowering mortality.^(5-9)^

The aim of the present study was to systematically review the main methods for nutritional risk assessment used in critically ill cancer patients, determining which methods better assess risks and predict relevant clinical outcomes in this group of patients and discussing the pros and cons of these methods according to the current literature.

## METHODS

The present study consisted of the analysis of references found in the following databases: PubMed (National Library of Medicine and National Institutes of Health - USA), LILACS, the comprehensive index of scientific database for Latin America and the Caribbean, and SciELO (Scientific Electronic Library Online), a cooperative digital database of open access journals originally from Brazil. To identify all relevant publications, we performed systematic searches in the reference databases for the last 20 years up to December 10, 2014. The search strategy was defined by keywords related to the assessment of nutritional risk and nutritional status terms (assessment of nutritional risk or nutritional status, nutritional assessment) in combination with ICU and critically ill patients with cancer terms [(critically ill (ness) cancer patient, critically ill (ness) cancer)].

Study selection and extraction of data (e.g., author, year, study sample, goal, nutritional assessment and main results) were performed simultaneously by two reviewers. Differences were resolved through a consensus procedure. In the event of disagreement, a decision was made by a third reviewer.

Manuscripts were assessed for the two main research questions: 1) the validity of a nutrition screening tool versus a reference method (criterion and construct validity); and 2) the ability of a tool to predict clinical outcome (predictive validity).^(10)^

Initially, a total of 34 articles were retrieved from PubMed, while the LILACS and SciELO databases did not provide any articles. Of these 34 articles, 2 were duplicates and were excluded from the analysis. Of the remaining 32 articles, we identified those that met the inclusion criteria. The inclusion criteria were previously defined as follows: 1) Studies in adults or the elderly; 2) written in English or Portuguese language; 3) studies performed in the general critically ill cancer patient hospital population (submitted or not to clinical or surgical treatments); 4) studies that described the predictive validity of a tool for one or more outcomes (nutritional status, length of stay, loss weight, malnutrition, overfeeding, mortality, survival, and other complications); 5) manuscripts published in the last 20 years. No articles in Portuguese were found. Papers were excluded if they met the following criteria: 1) were review articles, articles unavailable in full, short/brief communications and those not concerned with the nutritional assessment in critically ill cancer patients; 2) did not address the assessment of nutritional status in critically ill cancer patients; 3) did not address cancer; 4) were unavailable even upon request to the authors; 5) did not address the defined inclusion criteria.

Of the 32 articles identified in the search, 9 (28%) were excluded because they did not address the assessment of nutritional status in critically ill cancer patients, 4 (12%) did not address cancer, 5 (15%) did not meet the inclusion criteria, 2 (6%) were unavailable even upon request to the authors; 2 (6%) were short/brief communications, and the remaining 4 (12%) were review articles.

## RESULTS

**Figure 1:**
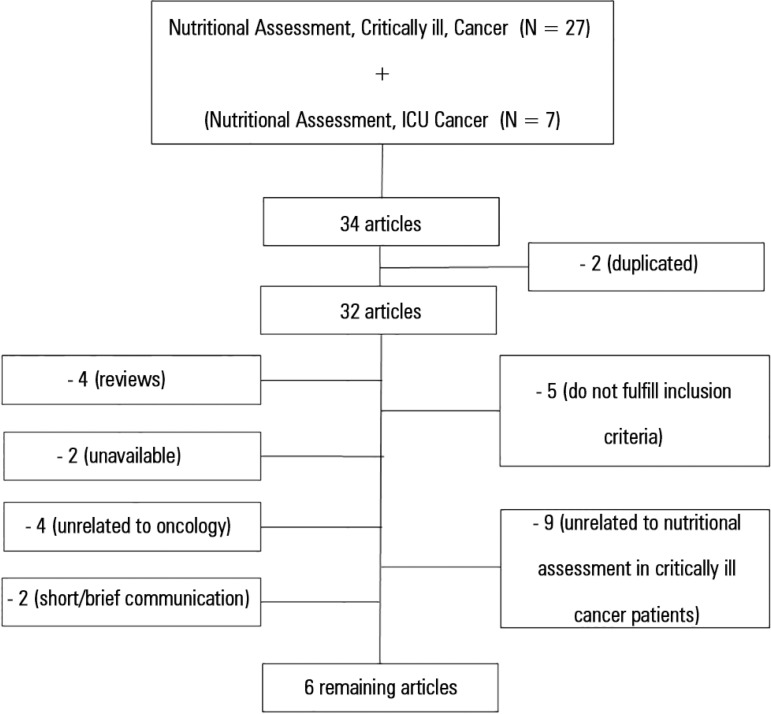
Schematic drawing of the methodology applied.

The flowchart describing the search for articles is presented in figure 1. Initially, 34 studies were selected, and in the end, only 6 articles (17.7%) were selected to compose this review. Articles are described in table 1.

**Table 1:** Indexed articles (PubMed, LILACS and SciELO) relating to nutritional assessment in critically ill cancer patients

**Author, year**	**Study, sample**	**Aim**	**Nutritional assessment**	**Main results**	**Final outcomes**
Pirat A et al., 2009^(11)^	Retrospective cross-sectional N = 34	Assess agreement between estimated REE (Harris-Benedict and clinical formula) vs. measured by indirect calorimetry	Indirect calorimetry, Harris-Benedict, Clinical formula, Weight and BMI	Significant correlation (p < 0.001) measured versus estimated REE (r = 0.587), with measured REE similar to estimated by Harris-Benedict without adding stress or activity factors	Undernourishment overfeeding
				Estimated methods associated with high incidence of malnutrition (90% REE) or overfeeding (110% REE)	
Khoshnevis N et al., 2012^(12)^	Prospective cross-sectional N = 416	Determine the prevalence and levels of malnutrition using the PG-SGA	PG-SGA	Prevalence of malnourishment: PG-SGA B = 29.1% and PG-SGA C = 24%	Low food intake weight loss malnutrition
				Strong correlation between PG-SGA versus Weight Loss (r = 0.684), clinical symptoms (r = 0.754) and nutritional symptoms (r = 0.801)	
				Nutritional symptoms were significantly related to reduced food intake (r = 0.652, p < 0.001) and weight loss (r = 0.577, p < 0.001)	
Salahudeen AK et al. 2009^(13)^	Retrospective observational N = 199	Examine predictors of survival outcomes	Urea, creatinine, albumin, weight and BMI	↑urea (≥ 8mg/dL) = lower risk of mortality (p = 0.03)	Mortality
				Higher levels of serum creatinine (RR - 0.8; 95%CI 0.66 -0.98) and serum albumin (RR - 0.68; 95%CI 0.51 - 0.92) = significantly lower risk of mortality (p = 0.03 and p = 0, 01) Less weight in the lowest serum urea (76 ± 21kg; p = 0.001)	
Read JA et al., 2006^(14)^	Follow-up study N = 51	Correlate survival and methods of assessing nutritional status	PS, CRP albumin, Weight Loss, ALP GPS, Weight, BMI and PG-SGA	Worst survival in poor ECOG-PS (p < 0.001), hypoalbuminemia (< 35g/L; p = 0.017), ↑ALP (p = 0.018), PG-SGA ≥ 9 (p < 0.001), PG-SGA B or PG-SGA C (p = 0.02) and GPS 1 or 2 (p = 0.036)	Mortality
				Significant negative correlation of CRP with survival (p = 0.029)	Survival
				Significant predictors of survival: Treatment (RR = 1.48; 95%CI = 1.11 to 1.79; p = 0.005) ECOG-PS (RR = 2.37; 95%CI = 1.11 to 5.09; p = 0.026) GPS (RR = 2.27; 95%CI = 1.09 to 4.73; p = 0.028) ALP (RR = 0.44; 95%CI = 0.18 to 1.07; p = 0.069) Nutritional status (NS)	
Lee JS et al., 2013^(16)^	Prospective observational N = 401 (N = 70 with metastatic cancer; N = 32 with non-metastatic cancer)	Validate GNRI as a predictor of hospital mortality in the short term (28 days)	GNRI, BMI, weight, albumin, CRP, creatinine	GNRI < 82 (p = 0.002) and 82 to < 87 (p = 0.015) = independent factor for increased risk of death versus GNRI > 98	Mortality
				Lower serum albumin associated with hospital mortality (cutoff < 3.5g/dL) (OR, 4.095; 95%CI, 2.219 - 7.557) (p < 0.001)	
				Cancer metastasis (p < 0.001) and serum creatinine levels (p = 0.011) associated with an increased risk of death	
Nelson and Walsh, 2002^(17)^	Prospective cross-sectional N = 50	Determine PINI	PINI	PINI normal value in a healthy population: < 1	Abnormal nutritional status
				Nutritional Status Assessment: ↑PINI = (SD) 102 (142) (95%CI of 62 - 142) in patients with advanced cancer, anorexia and weight loss	

REE - resting energy expenditure; BMI - body mass index; PG-SGA - Subjective Global Assessment Produced by the patients; RR - relative risk; 95%IC - 95% confidence interval; PS - performance status; CRP - C-reactive protein; ALP - alkaline phosphatase; GPS - Glasgow prognostic score; ECOG-PS - Eastern Cooperative Oncologic Group performance status; NS - not significant; GNRI - Geriatric Nutritional Risk Index; OR - odds ratio; PINI - prognostic inflammatory and nutritional index; SD - standard deviation.

**Figure 2:**
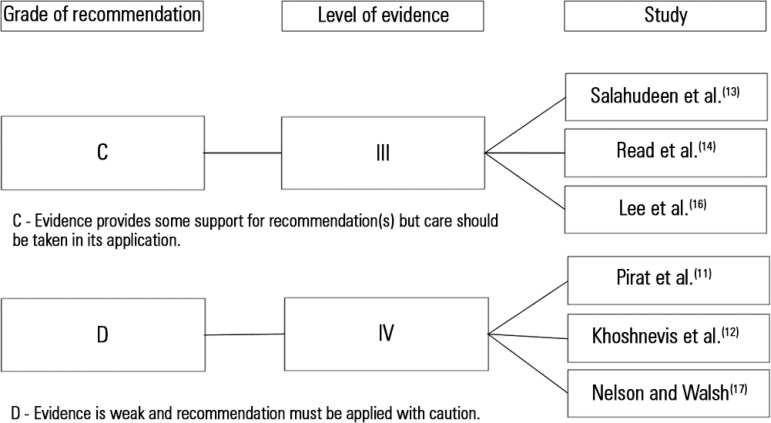
National Health and Medical Research Council additional levels of evidence and grades for recommendations for developers of guidelines.

Of the 6 selected articles, 3 were cross-sectional studies (n = 1 retrospective; n = 2 prospective), and 3 were observational studies (n = 1 retrospective; n = 2 prospective). Due to the differences in the studies included in this review, the authors defined the grade of recommendation and level of evidence for the articles following the National Health and Medical Research Council additional levels of evidence and grades for recommendations for developers of guidelines (Figure 2). The main outcomes observed in this review were undernourishment, overfeeding, nutritional status, mortality, survival, weight loss, weight and malnutrition.

Of all selected studies, 1 examined the correlation between estimated and measured resting energy expenditure (REE) to better estimate energy requirements; 1 assessed the prevalence of malnourishment in the general population by the Patient-Generated Subjective Global Assessment (PG-SGA); 1 examined the role of biochemical markers as predictors of mortality; 1 correlated mortality and survival outcomes with weight loss and different nutritional assessment methods, such as PG-SGA, ECOG-Performance Status (ECOG-PS), Glasgow Prognostic Score (GPS), biochemical markers and anthropometry; 1 validated the performance of the Geriatric Nutritional Risk Index (GNRI) in addition to analyzing biochemical markers as short-term predictors of mortality; and the last applied the Prognostic Inflammatory and Nutritional Index (PINI) to assess the nutritional status of the study population.

For better analysis and description of the results (Table 2), the articles selected for this review were separated according to the proposed methods of assessment.

**Table 2:** Nutritional assessment methods as predictors of nutritional risk in critically ill cancer patients

**Methods**	**Lee JS et al.^(16)^**	**Khoshnevis N et al.^(12)^**	**Salahudeen AK et al.^(13)^**	**Pirat A et al.^(11)^**	**Read JA et al.^(14)^**	**Nelson and Walsh^(17)^**
GNRI	+++	NA	NA	NA	NA	NA
PINI	NA	NA	NA	NA	NA	+++
PG-SGA	NA	+++	NA	NA	+++	NA
ECOG-PS	NA	NA	NA	NA	+++	NA
GPS	NA	NA	NA	NA	++	NA
Urea	NA	NA	++	NA	NA	NA
Creatinine	++	NA	++	NA	NA	NA
CRP	++	NA	NA	NA	++	+++
Albumin	+++	NA	++	NS	++	++
ALP	NA	NA	NA	NA	++	NA
Weight loss	NA	++	NA	NA	++	++
Weight	NS	NA	NS	NS	NS	NA
BMI	NS	NA	NS	NS	NS	NA
Harris-Benedict	NA	NA	NA	++	NA	NA
Clinical estimate	NA	NA	NA	NS	NA	NA
Indirect calorimetry	NA	NA	NA	+++	NA	NA

+ shortly significant;

++ significant;

+++ highly significant.

GNRI - Geriatric Nutritional Risk Index; NA - not available; PINI - prognostic inflammatory and nutritional index; PG-SGA - Subjective Global Assessment Produced by the patients; ECOG-PS - Eastern Cooperative Oncologic Group performance status; GPS - Glasgow prognostic score; CRP - C-reactive protein; ALP - alkaline phosphatase; BMI - body mass index; NS - not significant.

### Nutritional assessment based on indirect calorimetry and estimated methods

A retrospective cross-sectional study was conducted by Pirat et al.^(11)^ Of the 34 critically ill cancer patients included, 26 (76%) were postoperative. In the study, authors evaluated the correlation between the REE measured by indirect calorimetry with the estimated clinically REE by applying clinical formula (based on the American Society for Parenteral and Enteral Nutrition’s 2002 and 2004) and estimated REE by applying the Harris-Benedict equation. The REE estimated by Harris-Benedict equation, without including stress or activity factors, were similar to measured REE and exhibited a significant correlation (r = 0.587; p < 0.001), unlike the measured and clinically estimated REE (r = 0.24; p = 0.17). As the main outcomes, both the Harris-Benedict equation and clinically estimated formula were associated with high prevalence of malnutrition (29% and 15%, respectively) and overfeeding (29% Harris-Benedict equation and 71% clinical estimation method). Further studies are needed to permit assessment of the risks that overfeeding or underfeeding can create in this group of patients.

### Nutritional assessment based on subjective methods

Nutritional assessment by PG-SGA (Khoshnevis et al.) was performed in a prospective cross-sectional study with 416 critically ill cancer patients who were receiving surgery (18.0%), radiotherapy (31.8%), chemotherapy (32.8%) or a combination of these treatments (8.5%) or who were in their last treatment stages and follow-up care (9.0%). The authors analyzed the prevalence and levels of malnutrition in these patients. Well-nourished (A) patients made up 47% of the cases, those at nutritional risk (B) 29%, and 24% were severely malnourished (C). Considering the differences between treatments in cancer, malnourishment (grade B or C) was found in patients receiving surgery (21.5%), radiotherapy (32%), chemotherapy (30.6%), a combination of these (10%) or those in their last treatment stages and follow-up care (5.9%). Nutritional symptoms such as depression (39%), anorexia (38%), xerostomia (32%), nausea (25%) and pain (23%) were significantly related to reduced food intake (r = 0.652, p < 0.001) and weight loss (r = 0.577, p < 0.001). Of the 416 patients, 41% had no weight loss in the past 6 months and 44% had weight loss > 5% in 1 month or > 10% in 6 months. A strong correlation between weight loss and high PG-SGA (r = 0.684), clinical (r = 0.754) and nutritional (r = 0.801) symptoms was found.^(12)^

### Nutritional assessment based on laboratory parameters

Serum albumin, creatinine and urea were evaluated by Salahudeen et al.^(13)^ in a retrospective observational study that examined the predictors of survival outcomes in cancer patients (n = 199) treated with low-efficiency dialysis performed continuously (C-SLED). Twenty-two patients were receiving surgery, 20 radiotherapy and 86 chemotherapy. As the main outcomes, the study found that patients who had higher levels of urea (≥ 8mg/dL) demonstrated a significantly lower risk of death compared to those with lower levels of urea (< 8mg/dL; RR = 0.57; 95%CI from 0.34 to 0.94; p < 0.03). In contrast, patients who had higher levels of creatinine (RR = 0.8; 95%CI from 0.66 to 0.98) and serum albumin (RR = 0.68; 95%CI from 0.51 to 0.92) demonstrated a lower risk of death (p = 0.03 and p = 0.01). Body mass index (BMI) was not correlated (p = 0.18) with patient survival.

### Nutritional assessment based on combination of parameters

Read et al.^(14)^ conducted a follow-up study of 51 palliative-care patients with advanced stage IV colorectal cancer to correlate markers of nutritional assessment with survival. The markers included PG-SGA, Eastern Cooperative Oncologic Group - performance status (ECOG-PS), GPS, C-reactive protein (CRP), albumin, weight and BMI. Of the 51 patients in this study, 15 patients (29%) had recently been diagnosed with stage IV colorectal cancer, 36 (71%) had progressive disease after previously receiving one to three chemotherapy regimens, 37 patients (73%) had prior surgery, and 4 patients (8%) had previous radiotherapy.

The ECOG-PS is a subjective method that was primarily designed to assess the degree of clinical impairment caused by the tumor.^(15)^ In one study,^(14)^ 92% of patients had ECOG-PS 0-1 (without major limitations in daily activities), whereas only 8% of patients had ECOG-PS-2 (some limitations in daily activities). As the main outcome, poorer survival with worse ECOG PS was observed, as expected (p < 0.001). For PG-SGA, 56% of patients (n = 28) were classified as categories B or C. When the PG-SGA score was ≥ 9 (n = 19), 38% of patients were at high nutritional risk. Poor survival was observed in patients who had a PG-SGA score ≥ 9 (p < 0.001), PG-SGA B or PG-SGA C (p = 0.020). Weight loss ≥ 10% at 6 months was observed in 9 (18%) patients in the study.

In regard to biochemical tests, 33 (69%) patients had elevated CRP (> 10mg/L), 7 (14%) patients showed reduced albumin (< 35g/L), and 29 (57%) patients had high alkaline phosphatase (ALP) (> 130U/L). In the evaluation of GPS, a prognostic score that ranks inflammatory response based on a combination of CRP and albumin results, 7 (15%) patients were GPS 2 (hypoalbuminemia and increased CRP), 26 (54%) were GPS 1 (high PCR or hypoalbuminemia), and 15 (31%) were GPS 0 (no changes). As the main outcomes, poorer survival was observed in patients with hypoalbuminemia (< 35g/L; p = 0.017), high ALP (p = 0.018) and GPS 1 or GPS 2 (p = 0.036).

When CRP was assessed as a continuous variable rather than by category (normal versus high), a significant negative correlation with survival was found (p = 0.029). Multivariate survival analysis, using the Cox proportional hazard model, showed that type of treatment (RR = 1.48; 95%CI = 1.11 to 1.79; p = 0.005), ECOG-PS (RR = 2.37; 95%CI = 1.11 to 5.09; p = 0.026), GPS (RR = 2.27; 95%CI = 1.09 to 4.73; p = 0.028) and ALP (RR=0.44; 95%CI = 0.18 to 1.07; p = 0.069) could significantly predict survival. However, the nutritional status of patients was not significant predictor in the multivariate analysis.

In conclusion, authors suggested ECOG-PS, GPS, ALP and type of treatment are considered important predictors of survival in advanced colorectal cancer.

### Nutritional assessment-based risk-screening tools

Lee et al.^(16)^ conducted an observational study of septic patients 65 years and older (N = 401) to validate the performance of the GNRI, a screening tool for nutritional risk in predicting short-term hospital mortality (up to 28 days). Of the 401 patients studied, 70 (17.5%) had metastatic cancer, while 32 (8%) did not. Screening by GNRI and biochemical markers (albumin, CRP, creatinine) was performed for all patients. According to the GNRI, hospital mortality was 4.6% in the very low-risk group (GNRI > 98); 10% in the low-risk group (GNRI 92 - 98); 8.5% in the moderate risk group (GNRI 87 to < 92); 22% in the high-risk group (GNRI 82 to < 87); and 36% in the very high-risk group (GNRI < 82). The main outcomes were that GNRI less than 87, between 87 and 82 (p = 0.015) and < 82 (p = 0.002) were independently associated with an increased risk of death compared with GNRI > 98. Lower serum albumin was associated with hospital mortality (OR = 4.095; 95%CI = 2.22 to 7.56) after univariate analysis and failed to be significant after multivariate analysis (OR = 1.831; 95%CI = 0.78 to 4.28). Cancer metastasis (p < 0.001) and serum creatinine levels (p = 0.011) were the only independently factors associated with increased risk of death after multivariate analysis.

### Nutritional assessment based on scores

Nelson and Walsh conducted the first cross-sectional pilot study in palliative care of critically ill cancer patients (n = 50) using the PINI, which takes the product of two acute phase proteins (alpha-1 acid glycoprotein and PCR) divided by two visceral proteins (albumin and pre-albumin), and compared their PINI values with those of a healthy population (< 1). The average (SD) score in the sample was 102 (142), 95%CI = 62 - 142. No palliative patient was receiving active antitumor treatment. PINI was significantly higher in patients with advanced cancer, anorexia and weight loss and may be a useful method of assessment in critically ill cancer patients.^(17)^

The main results using the tools referenced above show that some methods of nutritional assessment proved to be better for clinical outcomes in critically ill cancer patients. Combined estimations of weight loss, serum measurements of CRP, albumin, urea, creatinine and alkaline phosphatase, preferably combined with other methods using scores such as ECOG-PS, GPS and PG-SGA, were associated with relevant clinical outcomes such as malnutrition, survival and mortality. It is interesting that both nutritional risk and status can be assessed by different methods to better estimate prevalence, prognosis and even response to nutritional interventions,^(5)^ which might reduce the risk of morbidity and mortality considerably.^(18)^

## DISCUSSION

More than 70 nutritional assessment tools have been described and analyzed in different populations. To date, no sufficiently sensitive and specific tool can be considered the gold standard for nutritional assessment.^(10)^ Fluid retention, tumor mass, chemotherapy side-effects such as hyperemesis, anorexia, fatigue and depression, liver, kidney or other organs toxicity, and supportive therapy effects leading to nausea and altering intestinal motility are among the conditions impairing assessment.^(19)^

Methods to estimate the energy needs of critically ill patients without cancer are generally inaccurate and often not feasible to perform in the ICU. Thus, to date none of these methods has been widely accepted. In critically ill cancer patients, Pirat et al. showed that malnutrition and overfeeding are common when these estimation methods are used,^(11)^ which can lead to unexpected outcomes.^(20)^

The PG-SGA applied in critical cancer patients, first by Read et al.^(14)^ and subsequently by Khoshnevis et al.,^(12)^ specifically addresses nutritional features of cancer patients and detects small variations in nutritional status.^(21)^ Read et al. found a high prevalence of malnutrition, nutritional risk and poor survival applying by the PG-SGA.^(14)^ Similarly, Khoshnevis et al. found that 50% of the studied patients had reduced food intake resulting from weight loss and consequent malnutrition, with almost half of the patients (46%) requiring intensive medical care. Nutritional symptoms, weight loss and reduction of fat and muscle tissue were considered factors causing malnutrition in such patients.^(12)^

Anthropometric parameters such as BMI, weight loss, muscle circumferences and skinfold thicknesses do not reflect the actual nutritional status when applied separately.^(6)^ Among the reviewed studies that examined weight and BMI alone, no clinical outcome was found.^(11,13,14,16)^ Weight loss combined with other parameters was strongly associated with high PG-SGA and nutritional symptoms.^(12)^ Thus, we suggest that nutritional assessment by anthropometric parameters, principally weight loss, should be performed in combination with assessment of other proposed parameters to obtain the best results.

Biochemical markers have gained considerable scientific and clinical value in recent years and are extremely useful throughout the disease process in combination with nutritional assessment. These assessments can be used to screen and assess risk, to determine the degree of nutritional damage and support type to be applied and to monitor the efficacy of nutritional support.^(22,23)^ Although these markers were considered useful and to be predictors of mortality for critically ill cancer patients by Salahudeen et al.^(13)^ and Read et al.,^(14)^ their results may be affected by disease-related factors and are not reliable indicators of malnutrition.^(4,24,25)^

The ECOG-PS is a subjective score designed to assess the degree of clinical involvement that the tumor imposes on the patient.^(15)^ Recently, Forrest et al. developed a new prognostic score known as the Glasgow Prognostic Score (GPS). GPS, a cumulative score based altered serum CRP and decreased albumin,^(26)^ is used to determine degree of inflammation but is also a potentially useful tool for nutritional assessment because cancer patients are considered to be in a constant state of chronic inflammation, which is one of the primary factors leading to cachexia. This score can also identify patients who may develop complications during treatment and is related to survival.^(27)^ Read et al.^(14)^ similarly reported that GPS and ECOG-PS were significant predictors of survival in critically ill patients with advanced colorectal cancer, and GPS features were similar to ECOG-PS prognostic values.^(26)^

The GNRI is a simple and objective screening tool for the nutritional risk assessment of in-hospital elderly patients and was first applied in critically ill cancer patients by Lee et al.^(16)^ This tool requires a single routine measurement of albumin, weight and knee height at admission and is not considered to be time consuming and demands little patient involvement. GNRI is also a more reliable prognostic indicator of hospital morbidity and mortality compared to albumin or BMI alone. However, the use of this score has several limitations because it can only be used in the elderly, and it is difficult to establish a normal weight in this population.^(16,28)^

The PINI was designed to assess both nutritional status and prognosis in critically ill patients because it can be used to track most pathological conditions.^(17,29)^ Nelson and Walsh concluded that there were no current methods that could accurately determine malnutrition in cancer and that the PINI could be helpful. Nevertheless, that pioneer study provided only preliminary information,^(17)^ and more studies applying PINI in critically ill cancer patients are needed to be able to compare data and outcomes in the future and possibly establish a recommendation for the use of the tool.

A systematic review including critically ill cancer patients was presented by Wong et al. in 2001. Although the focus of that review was nutritional support in critically ill cancer patients, the authors briefly discussed the importance of nutritional assessment in this group of patients. From the authors’ viewpoint, all proposed methods have a number of limitations for use in risk assessment and determining nutritional status. Therefore, no standardized recommendation can be provided yet.^(4)^

Recently, a systematic review to study construct or criterion validity and the predictive validity of nutrition screening tools for the general hospital population was presented. One of the limitations was the heterogeneity of the population. Therefore, the next step for future research would be to apply different tools in the same patient population, allowing for comparisons between tools and pooling of results. The authors reported that all 32 assessed tools showed inconsistent results with regard to construct validity. In conclusion, the group advised not developing any other assessment tools and never relying solely on a single tool to screen or assess patients’ nutritional status. In the absence of a recognized gold standard for the assessment of malnutrition, the research group considered assessment of anthropometric measures and the subjective global assessment to be ‘valid’ reference methods. Screening tools and laboratory values were thus considered less valid comparisons.^(10)^

We agree that nutritional risk assessment in critically ill cancer patients should be performed by combining distinct methods, considering all limitations and aiming to establish as reliable and complete a nutritional diagnosis as possible. However, in clinical practice, it is necessary to know the tools currently applied for the treatment of critically ill cancer patients and to discuss the pros and cons of these assessments to develop a new tool to assess the nutritional risk and nutritional status of this group.

This systematic review has some limitations. Only 6 studies assessed nutritional risk in cancer patients who had complications. Importantly, of the total of 6 articles included in this systematic review, 3 were pioneers in most methods of nutritional assessment in this group of patients,^(14,16,17)^ clearly demonstrating that there are no sufficient comparative studies. In addition, cancer patients were not divided into categories according to the anticancer treatment they were receiving to provide better assessment of nutritional risk. However, the instruments used in clinical practice do not consider the risks and complications of treatments in oncology, such as side effects of chemotherapy and radiotherapy and the implications of the postoperative inflammatory response.^(8)^

## CONCLUSION

Because no clear recommendation can yet be given regarding nutritional risk in critically ill cancer patients due to the lack of evidence from comparative studies, it is suggested that assessment should be performed by combining different methods and taking into account the limitations of each method. Considering the main outcomes using the various tools, methods of nutritional assessment should be based on combined estimations of weight loss, serum measurement of C-reactive protein, albumin, urea, creatinine and alkaline phosphatase, preferably along other methods using scores such as Eastern Cooperative Oncology Group performance status, Glasgow Prognostic Score and Patient-Generated Subjective Global Assessment.
